# Extracellular matrix specific protein fingerprints measured in serum can separate pancreatic cancer patients from healthy controls

**DOI:** 10.1186/1471-2407-13-554

**Published:** 2013-11-21

**Authors:** Nicholas Willumsen, Cecilie L Bager, Diana J Leeming, Victoria Smith, Morten A Karsdal, David Dornan, Anne-Christine Bay-Jensen

**Affiliations:** 1 Nordic Bioscience A/S, Biomarkers & Research, Herlev Hovedgade 207, DK-2730 Herlev, Denmark; 2 Gilead Sciences Inc., Biology, Foster City 94404, CA, USA

**Keywords:** Extracellular matrix remodeling, Collagen turnover, Biomarker, Serological detection, Matrix metalloprotease, Pancreatic cancer

## Abstract

**Background:**

Pancreatic cancer (PC) is an aggressive disease with an urgent need for biomarkers. Hallmarks of PC include increased collagen deposition (desmoplasia) and increased matrix metalloproteinase (MMP) activity. The aim of this study was to investigate whether protein fingerprints of specific MMP-generated collagen fragments differentiate PC patients from healthy controls when measured in serum.

**Methods:**

The levels of biomarkers reflecting MMP-mediated degradation of type I (C1M), type III (C3M) and type IV (C4M, C4M12a1) collagen were assessed in serum samples from PC patients (n = 15) and healthy controls (n = 33) using well-characterized and validated competitive ELISAs.

**Results:**

The MMP-generated collagen fragments were significantly elevated in serum from PC patients as compared to controls. The diagnostic power of C1M, C3M, C4M and C4M12 were ≥83% (p < 0.001) and when combining all biomarkers 99% (p < 0.0001).

**Conclusions:**

A panel of serum biomarkers reflecting altered MMP-mediated collagen turnover is able to differentiate PC patients from healthy controls. These markers may increase the understanding of mode of action of the disease and, if validated in larger clinical studies, provide an improved and additional tool in the PC setting.

## Background

Pancreatic cancer (PC) is an aggressive disease with a poor prognosis at time of diagnosis. It is estimated that roughly 80,000 people will die of the disease in 2013 in the EU and US [1,2]. At present, surgery is the only potentially curative therapy for PC as most tumors are unresponsive to basically all therapies [3]. Due to a lack of specific clinical symptoms, most patients have unresectable primary tumors and/or metastatic spread at the time of diagnosis, resulting in incurative disease. These facts make PC one of the top five most lethal types of cancer with approximately 70% of patients dying within the first year of diagnosis and with a five year survival rate at 5%. Consequently, there is an urgent medical need for biomarkers that can help making the diagnosis and prognosis of PC as well as assisting in monitoring recurrence of the disease and the response to treatment.

Among the different types of PC, pancreatic ductal adenocarcinoma (PDAC) accounts for almost 90% of cases [4]. In PDAC the stroma comprises approximately 80% of the tumor mass. The tumor stroma is characterized by the presence of cells such as fibroblast, endothelial cells and immune cells as well as by a dense and stiff extracellular matrix (ECM) referred to as desmoplasia. Desmoplasia is similar to fibrotic tissue and includes proteins such as collagens, fibronectin, proteoglycans and hyaluronic acid.

In many types of cancer, due to altered ECM remodeling from the desmoplastic reaction and an increased matrix metalloproteinase (MMP) activity [5], the tissue homeostasis is unbalanced leading to a different composition and quality of the ECM [6]. Especially, the interstitial fibrillar type I and III collagen [7], as well as the basement membrane protein type IV collagen [8,9] play important roles in tissue homeostasis alterations associated with PC. As a consequence of the altered ECM remodeling, ECM degradation fragments that contain specific neopeitopes, referred to as protein fingerprints, may be released to the circulation where they can be assessed and serve as surrogate markers of an altered ECM remodeling [10].

The aim of this study was to investigate whether protein fingerprints of specific MMP-generated collagen protein fragments may differentiate PC patients from healthy controls when measured in serum.

## Methods

### Patient serum samples

Patient serum samples were obtained from the commercial vendor Asterand (Detroit, MI). Asterand confirms that the following activities have been completed by their collaborators (as necessary): institutional and independent review board approval, privacy officer authorization, government licenses, or industry accreditations. All their informed consent templates were subject to review and approval by appropriate regulatory and ethics authorities. According to Danish law, it is not required to get ethical approval when measuring biochemical markers in previously collected samples; hence, there was no additional ethical approval for this particular study. The serum was collected from 15 patients with pancreatic ductal adenocarcinomas (PDAC) prior to resection, and 33 age- and sex-matched controls with no symptomatic or chronic disease (Table 1). Samples were collected, processed and stored in a similar fashion until analyzed, and all analyses were performed blindly. The study was conducted according to the Principal of Good Clinical Practice and according to the Declaration of Helsinki.

**Table 1:** Patient characteristics

**Group**	**No. of patients**	**Stage**					**Gender, % females**	**Age, years**
		**I**	**II**	**III**	**IV**	**Unknown**		**Mean ± SD (range)**
PDAC	15	3	8	1	-	3	40%	65 ± 8 (48 – 84)
Healthy controls	33						52%	61 ± 11 (43 – 78)

### ELISA measurements and procedure

Using well-characterized and validated competitive ELISAs, levels of MMP-2/-9/-13 degraded collagen type I (C1M) [11], MMP-9 degraded collagen type III (C3M) [12], MMP-9 degraded collagen type IV (C4M) [13] and MMP-12 degraded collagen type IV (C4M12a1) [14] were assessed in serum samples. The targets were identified from *in vitro* and *ex vivo* studies and by use of mass spectrometry (see references for details).

In brief, the individual serum biomarkers were assessed using a 96-well streptavidin-plate coated with a biotinylated specific synthetic peptide dissolved in an optimized assay buffer that was incubated for 30 minutes at 20°C. The plate was washed five times in washing-buffer (20 mM Tris, 50 mM NaCl, pH 7.2) prior to adding 20 μL of peptide calibrator or sample to the appropriate wells. This was followed by the addition of 100 μL of a HRP-conjugated target-specific monoclonal antibody. The plate was incubated for 1 hour at 20°C or overnight at 4°C, depending on the assay, and was then washed five times in washing-buffer. Finally 100 μL tetramethylbenzinidine (Kem-En-Tec cat.438OH) was added and the plate incubated for 15 minutes at 20°C in darkness before adding 100 μL stopping solution (1% H_2_SO_4_). The OD of each well was measured at 450 nm with 650 nm as reference.

### Statistical analysis

Biomarker levels from controls and patients were compared using an unpaired *t*-test on Log10 transformed data and are presented as Tukey box plots. Tumor stage was compared to controls by ANOVA. The area under the receiver operating characteristics (AUROC) was calculated for each biomarker and for all the biomarkers combined. Statistical analyses were performed using MedCalc Statistical Software v.12 (MedCalc Software, Ostend, Belgium). Results were considered statistically significant if p < 0.05.

## Results

### MMP-mediated degradation of collagen

**Figure 1:**
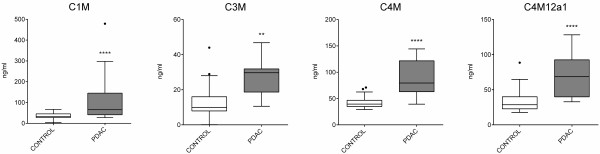
**Levels of MMP-generated fragments of type I, III and IV collagen are significantly elevated in serum from PC patients as compared to controls.** Biomarkers of MMP-2/-9/-13 mediated degradation of collagen type I (C1M), MMP-9 mediated collagen type III (C3M) and MMP-9 mediated (C4M) and MMP-12 mediated (C4M12a1) degradation of collagen type IV in serum from patients with pancreatic ductal adenocarcinomas (PDAC) (n = 15) and healthy controls (n = 33). Groups were compared using an unpaired *t*-test on Log transformed data. Results are presented as Tukey box plots, boundaries of each box indicate 25^th^ and 75^th^ percentiles, the line within the box marks the median and whiskers indicate maximum and minimum. Significance levels: **p < 0.01, ****p < 0.0001.

Levels of MMP-generated fragments of collagen type I, III and IV were significantly elevated in serum from PC patients as compared to controls (Figure 1). In detail, the level of MMP-2/-9/-13 degraded collagen type I (C1M) was 4-fold higher in PC patients as compared to controls and the levels of MMP-9 degraded collagen type III (C3M), MMP-9 degraded collagen type IV (C4M) and MMP-12 degraded collagen type IV (C4M12a1) were 2-fold higher in PC patients as compared to controls. Together, these findings indicate that altered collagen-remodeling is ongoing in PC.

**Figure 2:**
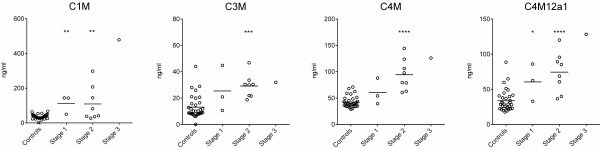
**Levels of MMP-generated fragments of type I (C1M), type III (C3M) and type IV (C4M and C4M12a1) collagen from PC patients as divided by tumor stage.** Biomarkers of MMP-2/-9/-13 mediated degradation of type I collagen (C1M), MMP-9 mediated type III collagen (C3M) and MMP-9 mediated (C4M) and MMP-12 mediated (C4M12a1) degradation of type IV collagen in serum from the patients (three of the patients had unreported stage) with pancreatic ductal adenocarcinomas (PDAC) and healthy controls. Stage 1 and 2 were compared to controls by ANOVA on Log transformed data. Significance levels: *p < 0.05, **p < 0.01, ***p < 0.01, ****p < 0.0001.

As the tumor stage is an important clinical tool in PC, the levels of MMP-generated fragments of type I (C1M), type III (C3M) and type IV (C4M and C4M12a1) collagen from PC patients as divided by tumor stage is illustrated in Figure 2. In detail, both patients in stage 1 and stage 2 had significantly elevated levels of C1M and C4M12a1 as compared to controls. The same was true for C3M and C4M, however only significant in stage 2 of the disease (clearly a trend was observed for elevated levels of these markers in stage 1 as well). Finally, the single patient in stage 3 was, for all markers tested except C3M, completely separated from the control group.

### Diagnostic power of biomarkers to discriminate between healthy and PC cases

The area under the receiver operating characteristic (AUROC) was calculated as a measure of the diagnostic power of the biomarkers individually as well as combined in healthy controls vs. PC (Table 2). The diagnostic power of C1M, C3M, C4M and C4M12a1 was highly significant with an AUROC ≥83% (p < 0.001). When combining all biomarkers, a diagnostic power of 99% (p < 0.0001) was achieved, indicating that the combination of all biomarkers leads to a complete discrimination between healthy controls and PC patients.

**Table 2:** Area under the receiver operating characteristic (AUROC) in discriminating between healthy controls and PC patients

**Biomarker**	**AUROC**	**Std. Error**	**95% confidence interval**	**P value**
C1M	0.83	0.067	0.701 to 0.963	<0.001
C3M	0.88	0.048	0.792 to 0.978	<0.0001
C4M	0.94	0.039	0.860 to 1.02	<0.0001
C4M12a1	0.89	0.046	0.805 to 0.985	<0.0001
All biomarkers combined	0.99	0.005	0.918 to 1.00	<0.0001

## Discussion

In this study we tested a panel of collagen degradation biomarkers in patients with pancreatic ductal adenocarcinomas (PDAC). Interestingly, significant differences in the biomarker levels were seen between healthy and diseased individuals. To our knowledge, this is the first study investigating the MMP-mediated turnover profile of several collagens in serum from PC patients.

The elevated levels of the biomarkers indicate that the normal homeostatic balance is changed in PC tissue. This may be caused both by increased local deposition and degradation of the ECM as desmoplasia (cancer associated fibrosis) which is a hallmark of PDAC [7], shares many similarities with general fibrosis where fragments of collagen type I [11], type III [12], and type IV [13] can be found in serum where it is linked to the ECM remodeling (production and degradation). In line with this desmoplasia is a dynamic process that contributes directly to malignancy through altered integrin signaling [15].

During PDAC progression the normal basement membrane architecture is lost and cancer cells have been found to be in direct contact with the interstitial matrix, i.e. type I and type III collagens [7]. Basement membrane protein alterations, such as collagen type IV degradation, have also been found to contribute to malignancy by enhancing the invasive behavior of pancreatic cancer cells [8]. Similar to our findings, collagen type IV has previously been found in serum of PC patients and linked (indirectly) to tissue remodeling [16]. Interestingly, in that study increased levels of circulating type IV collagen could be observed preoperatively, and postoperatively levels either increased or decreased with high levels corresponding to poor prognosis and short survival time. All together this indicates that MMP-driven remodeling of collagen type IV may be linked to severity and progression of the disease.

Elevated levels of circulating fragments of collagen type I, III and IV have been associated with different cancer types, including head and neck cancer [17], breast cancer [18,19], liver cancer [20], colorectal liver metastases [21] and for the hepatic failure following liver resection for hepatocellular carcinoma [22]. This indicates that collagen degradation and release of fragments to the circulation is an ongoing event in many types of cancer, and that collagen degradation markers may be applicable to disease monitoring in several cancer types. Novel information may be obtained from the neo-epitope based nature of the biomarkers applied in the current study. The neo-epitope technology is based on the concept that tumor associated proteases (MMPs) and tumor-tissue signature proteins (high levels of collagens) result in release of unique protein degradation fragments to the circulation. These protein fragments contain specific neoepitopes that may be more disease specific than measuring total protein and/or unspecific protein degradation fragments and may be indicative of a dynamic alteration in the tissue and thus contain important and novel information on mode of action of the disease. In the tumor microenvironment different proteases form complex proteolytic networks [23] and it is likely that different collagens and different proteases may be associated more or less with individual types of cancer.

The results presented here indicate that multiple MMPs are involved in the altered ECM-remodeling associated with PC. The mechanisms behind the degradation of the highly complex mixture of ECM proteins are still not fully understood. Different MMPs display diverse and sometimes opposite effects depending on tissue localization, cellular source, stage and cancer subtype [24,25]. MMPs are also very important in maintaining a healthy tissue homeostasis. This may also explain why many anti-MMP treatments tested so far have failed, i.e. harmful inhibition of the healthy MMP activity and unwanted inhibition of the anti-tumor effect of an increased MMP activity [26]. Furthermore, in a complex way degradation of ECM proteins such as collagen type IV may have both pro- and anti-tumorigenic effects. For instance, antibody-targeting denatured type IV collagen has been found to suppress angiogenesis and tumor growth in vivo [27,28], whereas the type IV collagen-derived protein fragments arrestin, canstatin and tumstatin are well described inhibitors of tumor angiogenesis [29]. In order to effectively target MMPs in cancer, a better understanding of both their primary and secondary pro- and anti-tumorigenic effects is required and it is well recognized that MMP inhibitor studies should include biomarkers that reflect activation or inhibition of specific MMPs in vivo [30]. We speculate that the neo-epitope based biomarkers could be optimal for monitoring the ability of either broad-spectrum or selective MMP inhibitors to exhibit anticancer effects and potentially monitor benefits and/or harms of anti-MMP treatment. Other MMP-specific neo-epitope based biomarkers may be developed that could contribute to this complex issue.

As the collagen degradation biomarkers were elevated in all stages of the disease, this indicates that the markers may be applied in the early onset of the disease. Furthermore, due to the intrinsic dynamic nature of the markers they may have the potential to identify a sub-population of patients with significant desmoplasia and invasion that may be fast progressing. The importance of identifying fast progressing patients as early as possible is emphasized by the increasing evidence supporting the idea that attenuating the desmoplastic reaction and tissue remodeling in general may help limit the progression of pancreatic cancer. In line with this, findings show that in patients with small tumors of ≤10-20 mm in size, and no lymph node or metastatic spread, surgical resection increased the 5-year survival rates to 30-75% [31], and retrospective computed tomography (CT) scans of patients >6 months prior to clinical detection of the disease showed that PC was, when present, found to be fully resectable [32]. Furthermore, as a dense stroma in PDAC has been linked to the formation of a barrier for chemotherapy exerting its effects, the biomarkers may potentially be able to separate chemotherapy responders from non-responders [7,33].

The biomarkers provided excellent diagnostic information, especially when combined (Table 2). To fully analyze the diagnostic applicability of the collagen degradation markers used here, other types of cancer and diseases such as pancreatitis should be included in the study. Other limitations include a small sample size, lack of patient information and the cross-sectional nature of the study. To date, no diagnostic examination has proven clinically practical for PC [34] and consequently there is a need to search for easily accessible, highly sensitive and specific diagnostic biomarkers. Blood based biomarkers that may serve as surrogates for tumor alterations and hereby avoid invasive diagnostic techniques are always preferred. The gold standard serum biomarker related to PC is CA19-9. Major limitations of this biomarker exist however, as only 65% of patients with resectable PC have elevated levels of CA19-9 [34] and false negative results occur in the Lewis negative blood type population [35]. Therefore, the American Society of Clinical Oncology also reports that CA 19–9 is inadequate for reliable PC diagnosis [36]. Many other markers have been extensively discussed and tested as serological markers of PC, however, none have demonstrated optimal sensitivity and diagnostic value for clinical utility [34,37]. A panel of markers reflecting different patho-physiological processes involved in different pancreatic diseases will almost certainly be required for diagnosis, prognosis and assessment of the efficacy of interventions. The collagen turnover biomarkers may potentially combine with other tools (such as a recently described 25 protein platform based on the role of the immune system in PDAC [38]) and add additional information that may improve PC outcome.

## Conclusion

PC is a field with an urgent need for biomarkers that can aid in the diagnosis of PC and contribute to prediction of tumor burden as well as the early identification of potential recurrence of the disease or lack of response to a given intervention. A panel of highly specific (neo-epitope based) serum biomarkers reflecting altered MMP-mediated collagen turnover differentiate PC patients from healthy controls. These markers may increase the understanding of mode of action of the disease and, if validated in larger clinical studies, provide an improved and additional tool in the PC setting.

## Competing interests

Willumsen N., Bager C.L., Leeming D.J., Karsdal M.A., and Bay-Jensen A.C. are employed at Nordic Bioscience A/S which is involved in the discovery and development of biochemical markers. Karsdal M.A. owns stocks at Nordic Bioscience. Smith, V. and Dornan, D. are employed at Gilead Sciences Inc. involved in drug development.

## Authors’ contributions

NWI and CBA carried out the measurements, data analysis and performed the statistical analysis. NWI, CBA, MK, ACBJ, VS and DD participated in the design of the study. VS and DD provided the samples and patient information. NWI drafted the manuscript with help from CBA, DJL, MK and ACBJ. All authors read and approved the final manuscript.

## Pre-publication history

The pre-publication history for this paper can be accessed here:

http://www.biomedcentral.com/1471-2407/13/554/prepub
